# Supplementary material for: SARS-CoV-2 impairs male fertility by targeting semen quality and testosterone level: A systematic review and meta-analysis
Source: PLoS One. 2024 Sep 9;19(9):e0307396. doi: 10.1371/journal.pone.0307396 (PMC11383251; doi:10.1371/journal.pone.0307396)
Supplement: Attachment — Submitted filename: Response to Reviewers Comments_PlosOne.doc [file pone.0307396.s003.doc]

When submitting your revision, we need you to address these additional requirements.

1. Please ensure that your manuscript meets PLOS ONE's style requirements, including those for file naming. The PLOS ONE style templates can be found at
   <https://journals.plos.org/plosone/s/file?id=wjVg/PLOSOne_formatting_sample_main_body.pdf> and 
   <https://journals.plos.org/plosone/s/file?id=ba62/PLOSOne_formatting_sample_title_authors_affiliations.pdf>

**Response: Thanks. This has been ensured.**

2. We noticed you have some minor occurrence of overlapping text with the following previous publication(s), which needs to be addressed:

<https://www.frontiersin.org/journals/endocrinology/articles/10.3389/fendo.2023.1227836/full>

In your revision ensure you cite all your sources (including your own works), and quote or rephrase any duplicated text outside the methods section. Further consideration is dependent on these concerns being addressed.

**Response: Thanks. Overlapping phrases have been modified.**

3. Please provide a complete Data Availability Statement in the submission form, ensuring you include all necessary access information or a reason for why you are unable to make your data freely accessible. If your research concerns only data provided within your submission, please write "All data are in the manuscript and/or supporting information files" as your Data Availability Statement.

**Response: Thanks. The Data Availability Statement is included as “All data are in the manuscript and/or supporting information files”.**

4. Please include your tables as part of your main manuscript and remove the individual files. Please note that supplementary tables (should remain/ be uploaded) as separate ""supporting information"" files

**Response: Thanks. The Tables have been added as part of the main manuscript.**

5. Please include captions for your Supporting Information files at the end of your manuscript, and update any in-text citations to match accordingly. Please see our Supporting Information guidelines for more information: <http://journals.plos.org/plosone/s/supporting-information>.

**Response: Thanks.**

6. Please review your reference list to ensure that it is complete and correct. If you have cited papers that have been retracted, please include the rationale for doing so in the manuscript text, or remove these references and replace them with relevant current references. Any changes to the reference list should be mentioned in the rebuttal letter that accompanies your revised manuscript. If you need to cite a retracted article, indicate the article’s retracted status in the References list and also include a citation and full reference for the retraction notice.

**Response: Thanks. The references have been checked for appropriateness.**

**Additional Editor Comments:**

This is an interesting paper describing the effects of a Covid infection on the male reproductive system. Both referees are very positive but have some suggestions for minor revsion.

**Response: Thanks. All concerns raised have been addressed.**

[Note: HTML markup is below. Please do not edit.]

Reviewers' comments:

Reviewer's Responses to Questions

**Comments to the Author**

1. Is the manuscript technically sound, and do the data support the conclusions?

The manuscript must describe a technically sound piece of scientific research with data that supports the conclusions. Experiments must have been conducted rigorously, with appropriate controls, replication, and sample sizes. The conclusions must be drawn appropriately based on the data presented.

Reviewer #1: Yes

Reviewer #2: Yes

2. Has the statistical analysis been performed appropriately and rigorously?

Reviewer #1: Yes

Reviewer #2: Yes

3. Have the authors made all data underlying the findings in their manuscript fully available?

The [PLOS Data policy](http://www.plosone.org/static/policies.action#sharing) requires authors to make all data underlying the findings described in their manuscript fully available without restriction, with rare exception (please refer to the Data Availability Statement in the manuscript PDF file). The data should be provided as part of the manuscript or its supporting information, or deposited to a public repository. For example, in addition to summary statistics, the data points behind means, medians and variance measures should be available. If there are restrictions on publicly sharing data—e.g. participant privacy or use of data from a third party—those must be specified.

Reviewer #1: Yes

Reviewer #2: Yes

4. Is the manuscript presented in an intelligible fashion and written in standard English?

PLOS ONE does not copyedit accepted manuscripts, so the language in submitted articles must be clear, correct, and unambiguous. Any typographical or grammatical errors should be corrected at revision, so please note any specific errors here.

Reviewer #1: Yes

Reviewer #2: Yes

5. Review Comments to the Author

Please use the space provided to explain your answers to the questions above. You may also include additional comments for the author, including concerns about dual publication, research ethics, or publication ethics. (Please upload your review as an attachment if it exceeds 20,000 characters)

Reviewer #1: Dear Authors,
Congratulations for this interesting piece of research.
Some items worth considering:
1. It might be worth mentioning that duration of the infection and time between infection and semen collection might have an effect on the study outcomes. Based on current literature and on textbook knowledge, high fever might have a transient effect on semen analysis which might (or might not) be temporary.

**Response: Thanks. This has been included.**

2. Likewise, it might be worth mentioning if the studies were performed in early 2020, after introduction of COVID-19 vaccines, or following infection by the most recent and less dangerous variants of COVID-19.

**Response: Thanks. This has been included.**

3. Some of the studies were done before the release of the 2021 WHO manual. It might also be worth highlighting this difference, since it can affect the interpretation of the results, and maybe consider this when performing subgroup analyses.

**Response: Thanks. The WHO guideline would not affect the interpretation since the infective state was compared with a control (either COVID negative or PreCOVID or after treatment) and not just with the WHO standards.**

Reviewer #2: The manuscript to be assessed is a systematic review and meta-analysis of the influence of SARS-CoV-2 infection on male fertility. There are already meta-analyses on this topic, but the authors explain very well that, in their view, there are methodological weaknesses in the already published meta-analyses that they have explicitly addressed in their work.
Overall, this is a methodologically very well conducted meta-analysis. The methodological steps and statistical analyses carried out are very well documented and openly communicated. The associated illustrations, especially the forest plots, are very detailed and comprehensive, which seems necessary due to the large number of parameters analysed.

**Response: Thanks.**

Overall, in my view, this is a very mature manuscript for which I would like to congratulate the authors. The lack of originality in terms of existing publications is addressed by the authors and clearly explained what the methodological changes are here. As a suggestion for improvement, I would only add 2 aspects to the discussion.
1) I miss the discussion of the longitudinal aspect here, i.e. the duration of a potentially negative influence of SARS-CoV-2 infection on the reproductive functions of men, also with regard to the control intervals of the underlying studies.

**Response: Thanks. This has been included as suggested.**

2) in my view, it should also be discussed to what extent a SARS-CoV-2 infection represents a special feature compared to other systemically active viral infections, some of which are very severe (e.g. influenza), or whether this is not to be expected independently of the virus type.

**Response: Thanks. This has been included as suggested.**

6. PLOS authors have the option to publish the peer review history of their article ([what does this mean?](https://journals.plos.org/plosone/s/editorial-and-peer-review-process#loc-peer-review-history)). If published, this will include your full peer review and any attached files.


If you choose “no”, your identity will remain anonymous but your review may still be made public.


**Do you want your identity to be public for this peer review?** For information about this choice, including consent withdrawal, please see our [Privacy Policy](https://www.plos.org/privacy-policy).

Reviewer #1: No

Reviewer #2: No
